# Sudden occurence of hypotension and bradycardia during greenlight laser transurethral resection of prostate: case report of two cases

**DOI:** 10.1186/s12871-016-0234-x

**Published:** 2016-08-30

**Authors:** Zheng Guan, Jingjie Liu

**Affiliations:** 1 Department of Anesthesiology, The First Affiliated Hospital of Xi’an Jiaotong University, Yanta West Road, No.277, 710061 Xi’an, China; 2 Department of Neurology, The Second Affiliated Hospital of Xi’an Jiaotong University, Xi’an, China

**Keywords:** Parasympathetic reflex, Transurethral resection of prostate, Greenlight laser

## Abstract

**Background:**

Greenlight laser transurethral resection of prostate (TURP) is a standard surgical method used to treat patients with prostate gland enlargement, it is safe and effective.

**Case presentation:**

We report two cases of sudden occurence of hypotension and bradycardia during greenlight laser TURP. Two patients with benign prostatic hypertrophy were scheduled for greenlight laser TURP under spinal anesthesia. Hypotension and bradycardia were suddenly occurred during the operation. The blood gas analysis revealed no hyponatremia (indicating TURP syndrome) or anemia (indicating hemorrhage). Operation was suspended and inotropic agents were administrated intravenous immediately, then blood pressure and heart rate increased to normal level within some minutes. The patients were discharged from hospital without any complications. We considered parasympathetic reflex was occurred during greenlight laser TURP.

**Conclusion:**

Apart from TURP syndrome, hemorrhage, bladder perforation and high spinal anesthesia, the parasympathetic reflex which is caused by operative process can also induce hypotension and bradycardia during TURP.

## Background

Greenlight laser transurethral resection of the prostate (TURP) is a standard surgical method used to treat patients with prostate gland enlargement. Hypotension and bradycardia are complications of TURP, the primary causes are TURP syndrome, hemorrhage and bladder perforation [1, 2], high spinal anesthesia is also a common cause [3]. But we report other two cases who presented sudden and severe hypotension and bradycardia during greenlight laser TURP which wasn’t due to reasons above, but due to parasympathetic reflex. To the best of our knowledge, this is the first report that parasympathetic reflex can cause hypotension and bradycardia during greenlight laser TURP. We suggest that the strict monitoring of vital signs should be performed and the vasoactive drugs should be prepared during TURP, the heart rate and blood pressure should be maintained at a higher level during TURP, and the decline of heart rate and blood pressure should be intervened immediately. This study has been granted an exemption from requiring ethics approval according to “The ethics committee of the first affiliated hospital of Xi’an jiaotong unversity”.

## Case presentation

### Case one

A 78-year-old man underwent TURP due to benign prostatic hyperplasia, there were no coexisting diseases. The operation was performed under spinal anesthesia with 2 ml 0.5 % hyperbaric bupivacaine and at lithotomy position. The sensory block level was extended to the T10. The heart rate (HR) was 72 bpm and blood pressure (BP) was 140/75 mmHg at the start of the operation, the SpO2 was 100 % and the electrocardiograph (ECG) was normal. The TURP was performed by greenlight laser instrument. The irrigation fluid was 0.9 % normal saline. A hour later, when the operation site focused on the apex of prostate, the HR suddenly decreased to 38 bpm, the BP dropped to 68/36 mmHg, patient appeared nausea and pallor. The operation was suspended immediately, the 1 mg atropine was administrated intravenous, but the HR was not rised, then the dopamine was continuous infusioned at a dose of 5–10 ug.kg^−1^.min^−1^ after a bolus of 1 mg intravenous. The BP and HR slowly increased and returned to normal 30 min later. The blood gas analysis revealed the serum sodium was 138.1 mmol/l, the Hct was 27 % (28.4 % preoperation), the acid-base balance was normal. The operation was ended after tight hemostasis, the patient was recovered uneventful and discharged from hospital 7 days later in a good condition.

### Case two

A 65-year-old man underwent TURP due to benign prostatic hyperplasia, he was administrated a PCI due to acute cardiac infarction 5 years ago. The ultrasonic cardiography (UCG) indicated the eject fraction (EF) was 45 %, the cardiac function was III degree according to the New York Heart Association (NYHA). The operation and anesthesia were performed as same as the case one. The HR was 75 bpm and BP was 116/72 mmHg at the start of the operation, the SpO2 was 100 % and the ECG was normal. 40 min later, when the operation site focused on the apex of prostate, the HR suddenly decreased to 32 bpm, the BP dropped to 60/35 mmHg, patient became restless and agonal. The operation was suspended and the 0.5 mg atropine was administrated intravenous immediately, he was intubated and ventilated with an FiO2 of 60 % after general anesthesia induction(0.1 mg fentanyl, 15 mg etomidate, 50 mg rocuronium, sequential), invasive blood pressure monitor was performed through brachial artery caheter. Epinephrine was continuous infusioned at a dose of 0.03–0.1 ug.kg^−1^.min^−1^. The BP and HR increased to 102 bpm and 140/79 mmHg respectively five minutes later. The blood gas analysis revealed the serum sodium was 140 mmol/l, the Hct was 33 % (36 % preoperative), the acid-base balance was normal. The ECG was identified that acute myocardiac ischemia and acute coronary syndrome were not occured. The operation was ended after tight hemostasis, the patient was recovered in postanesthesia care unit (PACU) and extubated 30 min after operation, he made an uneventful recovery and was discharged from hospital 9 days later in a good condition. The arterial blood gas analysis and electrolyte levels of the two patients were displayed in Table 1.

**Table 1:** The arterial blood gas analysis and electrolyte levels of the two patients

Patient	Case one		Case two	
	T1	T2	T1	T2
pH	7.377	7.377	7.405	7.418
PCO2(mmHg)	40.5	42.6	40.8	39.6
PO2(mmHg)	154.1	119.8	114	346^②^
Hct(%)	27^①^	28.4	33	30
K+(mmol/l)	3.23	3.18	4.3	4.2
Na + (mmol/l)	138.1	139.2	140	138
HCO3(mmol/l)	24	25.2	25.2	25.4
BE(mmol/l)	−1.4	−0.2	0.9	1.1
Glucose(mmol/l)	10.9	7.71	8.2	5.2
Lactate(mmol/l)	2.8	0.8	1.6	0.8

*T1* The time point when hypotension and bradycardia occured, *T2* the time point when the patients’ condition became stable, ①: preoperative Hct is 28.4 %; ②: mechanical ventilated with FiO2 of 60 %

## Discussion

Hypotension and bradycardia were common complications during TURP, the reason of these complications are TURP syndrome, hemorrhage, bladder perforation and high spinal anesthesia. The TURP syndrome is a systemic complication caused by excessive absorption of electrolyte-free irrigation fluids [4], the incidence is 0.78–1.4 % [2]. The incidence of bladder perforation is 1 % during TURP [5]. We reported other two cases who presented sudden severe hypotension and bradycardia during greenlight laser TURP which weren’t due to TURP syndrome, hemorrhage or bladder perforation and high spinal anesthesia, but due to parasympathetic reflex.

We suspected it was parasympathetic reflex that lead to hypotension and bradycardia in these two patients. First, the hypotension and bradycardia occured suddenly, the symptom was similar to the classic vagal reflex caused by pulling reaction of viscera during appendectomy under epidural anesthesia [6]. Second, the symptom was improved when the operation was suspended and the atropine and inotropic drugs were used, they were also as same as the treatment of vagal reflex. Third, the symptom was occured when the same operative process was performed. It could be explained by a neural reflex.

The prostate anatomic position also related to parasympathetic activity, because prostate is surrounded by autonomic nerve fibers including sympathetic and parasympathetic nerves. The parasympathetic nervous center is located at S2-S4 section of spinal cord. The peripheral part is composed of inferior hypogastric plexus and pelvic splanchnic nerve. In which, pelvic splanchnic nerve is closer to the apex of prostate at the level of the membranous urethra. Besides, the parasympathetic nerves were mainly located dorsolaterally at the apex according to immunohistochemical analysis [7, 8].

Hypotension and bradycardia are common adverse effects of spinal anesthesia, the incidence is 15–38 % and 10 % respectively [3]. They usually occur in elder patients [9] and they usually occur at the beginning 15 min of intrathecal injection [10]. High spinal block (above T5) [11, 12] and high dose of heavy bupivacaine [12] are main anesthetic factors. But in the two cases we reported here, the hypotension and bradycardia occurred 40-60 min after intrathecal injection. And the spinal puncture was performed at the L3-4 intervertebral space, the level of sensory block was below T10 throughout the operation. The dose of bupivacaine was well controlled. So we think hypotension and bradycardia in our cases were caused by parasympathetic reflex instead of high spinal anesthesia.

The hypotension and bradycardia in our cases were not caused by TURP syndrome. First, TURP syndrome can cause hypervolemia and hyponatraemia [4], but it is rare when normal saline is used as irrigation fluid [13]. In our cases, normal saline is used as irrigation fluid, and there is no manifestation of hypervolemia and hyponatraemia. Second, TURP syndrome can cause a wide variety of symptoms including asymptomatic hyponatremia, ECG changes, fatigue, vomiting, confusion, visual loss, coma and death [14, 15]. So the change of mental state may be the first sign, but it was not appeared in our cases. Third, we didn’t give any treatment of TURP syndrome such as sedation, diuresis and sodium supplement [16], the cases recovered. Finally, TURP syndrome is defined as serum sodium concentration ≤ 125 mmol/l [17], but it was normal in our cases.

The intravesical explosion during TURP can cause bradycardia and hypotension, accompany with nausea, abdominal pain and developed confusion [1]. But this complication usually manifested as a “pop” and can be identified by the urologists and anesthesiologists. The bladder perforation can be observed through cystoscope. So the hypotension and bradycardia in our cases were not caused by bladder perforation.

Because the Hct was stable during operation, the hypotension and bradycardia were not caused by hemorrhage.

## Conclusion

Apart from TURP syndrome, hemorrhage, bladder perforation and high spinal anesthesia, parasympathetic reflex during TURP is another reason which can cause hypotension and bradycardia. We suggest that the strict monitoring of vital signs should be performed and the vasoactive drugs should be prepared during TURP. Don’t forget the possibility of parasympathetic reflex when sudden hypotension and bradycardia appeared in TURP.

## Abbreviations

BP: Blood pressure
bpm: Beat per minute
ECG: Electrocardiogram
EF: Ejection fraction
Hct: Haematocrit
HR: Heart rate
NYHA: New York Heart Association
PACU: Post anesthesia care unit
PCI: Percutaneous coronary intervention
TURP: Transurethral resection of prostate
UCG: Ultrasound echocardiography
